# Astaxanthin prevents osteoarthritis by blocking Rspo2-mediated Wnt/β-catenin signaling in chondrocytes and abolishing Rspo2-related inflammatory factors in macrophages

**DOI:** 10.18632/aging.204837

**Published:** 2023-06-23

**Authors:** Chunhui Zhu, Gang Liu, Weiding Cui, Zhongjie Yu, Wei Chen, Yao Qin, Jiuxiang Liu, Yaojun Lu, Weimin Fan, Wenwei Liang

**Affiliations:** 1 Trauma Center, The Affiliated Changzhou No.2 People’s Hospital of Nanjing Medical University, Changzhou 213003, China; 2 Department of Orthopedics, The First Affiliated Hospital of Nanjing Medical University, Nanjing 210029, China; 3 Department of Endocrinology, The First Affiliated Hospital of Nanjing Medical University, Nanjing 210029, China

**Keywords:** astaxanthin, Rspo2, macrophages, chondrocytes, coculture

## Abstract

Chondrocyte degeneration and classically activated macrophage (AM)-related inflammation play critical roles in osteoarthritis (OA). Here, we explored the effects of astaxanthin and Rspo2 on OA *in vitro* and *in vivo*. We observed that the Rspo2 gene was markedly elevated in synovial tissues of OA patients compared with healthy controls. In 2D cultures, Rspo2 and inflammatory factors were enhanced in AMs compared with nonactivated macrophages (NMs), and the protein expression levels of Rspo2, β-catenin, and inflammatory factors were increased, and anabolic markers were reduced in osteoarthritic chondrocytes (OACs) compared to normal chondrocytes (NCs). Astaxanthin reversed these changes in AMs and OACs. Furthermore, Rspo2 shRNA significantly abolished inflammatory factors and elevated anabolic markers in OACs. In NCs cocultured with AM, and in OACs cocultured with AMs or NMs, astaxanthin reversed these changes in these coculture systems and promoted secretion of Rspo2, β-catenin and inflammatory factors and suppressed anabolic markers compared to NCs or OACs cultured alone. In AMs, coculture with NCs resulted in a slight elevation of Rspo2 and AM-related genes, but not protein expression, compared to culture alone, but when cocultured with OACs, these inflammatory mediators were significantly enhanced at both the gene and protein levels. Astaxanthin reversed these changes in all the groups. *In vivo*, we observed a deterioration in cartilage quality after intra-articular injection of Rspo2 associated with medial meniscus (DMM)-induced instability in the OA group, and astaxanthin was protective in these groups. Our results collectively revealed that astaxanthin attenuated the process of OA by abolishing Rspo2 both *in vitro* and *in vivo*.

## INTRODUCTION

Osteoarthritis is a chronic degenerative disease characterized by degeneration and destruction of articular cartilage, accompanied by joint pain, swelling, deformity and mobility impairment, which seriously affects the quality of life of patients [1–3]. Osteoarthritis (OA) is characterized by cartilage damage, synovial inflammation, subchondral bone remodeling and osteophyte formation [3–5]. Under conditions of inflammation stimulation, chondrocytes, the main component of cartilage, degenerate due to the release of metalloproteinases and ADAMTS, which are responsible for the destruction of cartilage extracellular matrix and secretion of inflammatory factors such as IL-1β and TNF-a, exacerbating the development of OA [6, 7]. Therefore, maintaining the function of chondrocytes in an inflammatory state, reducing the secretion of inflammatory factors, and diminishing the destruction of the extracellular matrix is essential for the treatment of osteoarthritis.

In recent years, increasing evidence has confirmed that osteoarthritis is a low-grade inflammation affecting the entire joint, and synovitis has been widely confirmed to be closely related to the development of arthritis [8, 9]. Roemer et al. followed 514 normal knee joints for 30 months and found that diffuse synovitis was directly related to cartilage degeneration. Aryal et al. studied 422 patients with Kellgren Lawrence scores of 2~3 grades with MRI for 1 year and found that the degree of synovitis could predict the prognosis of osteoarthritis [10, 11]. The synovium is divided into two layers, the intimal lining layer and the synovial sublining layer, and macrophages residing in the intimal lining layer play central roles in the process of synovitis. Macrophage accumulation in the intimal lining layer is activated in the inflammatory environment, releasing inflammatory factors, chemokines and other factors that contribute to the destruction of cartilage [12, 13]. Therefore, regulating the functions of macrophages in the synovium and synovial fluid of patients with OA is crucial to the treatment of osteoarthritis. Macrophages can polarize to the M1 (classically activated macrophages) or M2 (alternatively activated macrophages) phenotype different microenvironmental stimuli [14, 15]. M1 macrophages can be activated by LPS and IFN-γ, releasing a large number of proinflammatory factors, such as IL-1β, TNF-α, and IL-6, to promote synovial inflammation. M2 macrophages, generally known as damage repair macrophages, can also be activated by IL-4, secreting anti-inflammatory factors such as IL-4, IL-10, and IL-13, which exert anti-inflammatory effects in various settings [16–18]. Increasing evidence has revealed that M1 macrophages play dominant roles in the onset and development of osteoarthritis [19, 20]. Dai et al. found that squid type II collagen can reduce synovial inflammation and osteoarthritis cartilage degeneration in rats by inhibiting the secretion of IL-1β and TNF-α by M1 macrophages [19]. Park et al. found that Tabetri could delay the progression of arthritis by inhibiting the expression of pro-inflammatory factors such as IL-1β and IL-6 of M1 macrophages in the serum of arthritic mice [20]. Therefore, blocking the polarization and reducing the secretion of inflammatory factors by M1 macrophages appear central for the treatment of osteoarthritis.

Astaxanthin is a hydrocarbon composed of 40 carbon atoms containing two terminal ring systems connected by conjugated double bond chains or polyene systems (C_40_H_52_O_4_) [21]. Astaxanthin, as a unique bioactive substance, exerts multiple functions, including antioxidation, anti-inflammation, and anti-apoptosis [22–24]. Due to its powerful bioactivity and safety, astaxanthin has been approved by the FDA as a food additive and is widely used by athletes as a nutraceutical [21, 25]. Astaxanthin has been widely confirmed to alleviate chronic and acute inflammation in various diseases, including neurodegenerative disorders, diabetes, gastrointestinal disease, renal inflammation, and skin and eye diseases [26]. However, few studies have focused on whether astaxanthin is essential for function of chondrocytes and M1 macrophages in the development of osteoarthritis and related regulatory mechanisms. Park et al. demonstrated that as a mixture contain astaxanthin, FP-MD treatment significantly blocked the process of OA in rats [27]. Another study by Stonehouse et al. revealed that Krill oil, rich in astaxanthin, was able to alleviate knee pain and stiffness, and improve physical function in adults with mild to moderate knee OA [28]. Intra-articular injection of astaxanthin was also reported to prevent cartilage damage in OA New Zealand rabbits [29]. Several researchers have confirmed the effects of astaxanthin on macrophages in some settings rather than OA. In the treatment of NASH, astaxanthin were proved to diminish the recruitment and activation of T-cells and macrophages/Kupffer cells, and promote an M2-dominant shift in macrophages/Kupffer cells to control inflammation and insulin sensitivity [30]. Work from Li revealed that astaxanthin played new anti- atherogenic properties by reducing macrophage infiltration, and modulating MMP3 expression in hyperlipidemic rabbits [31]. Based on the former evidence, we speculate that astaxanthin may inhibit the degeneration of chondrocytes and the polarization of M1 macrophages, thereby delaying the development of osteoarthritis.

As a member of the R-spondin family, Rspo2 has been shown to be associated with many pathological diseases [32, 33]. However, the specific effects of Rspo2 on the process of OA remain obscure. Several lines of evidence indicate that there may be some relationships between Rspo2 and osteoarthritis. Work from Lee explored the spatial expression of RSPO2 in early and advanced stage of human OA samples, and RSPOs played important roles in the interaction between chondrocytes and osteoblasts in the process of OA [34]. Okura and coworkers have observed that compared to normal controls, Rspo2 protein expression in synovial fluid from OA patients was elevated [35]. Rspo2 was also confirmed to be essential for altered osteogenesis or bone remodeling in OA osteoblasts in the progression of OA [36]. In addition, much evidence has revealed that the Wnt/β-catenin pathway plays a marked role in the process of OA, and inhibition of the Wnt/β-catenin pathway can attenuate the development of OA [37–39]. Rspo2 is widely accepted to play important roles in the activation of the Wnt/β-catenin signaling pathway. Based on this evidence, we speculated that Rspo2 may be a decision-maker in the treatment of OA. In the context of the participation of astaxanthin and Rspo2 in the process of OA, we speculated that there may be some relationship between astaxanthin and Rspo2. From the literature, no relationship between astaxanthin and Rspo2 has been studied. Several researchers have explored whether astaxanthin can reverse adverse effects by abolishing the Wnt/β-catenin pathway in some diseases, including oral cancer and hepatocellular carcinoma [40, 41]. As Rspo2 was proven to be an activator of the Wnt/β-catenin pathway, we speculated that astaxanthin may be an upstream regulator of Rspo2 in this setting.

Despite the complex process of osteoarthritis, the paracrine functions of macrophages and chondrocytes seem indispensable. When OA occurs, activated M1 macrophages release a variety of proinflammatory factors, such as IL-1β, IL-6, and TNF-α, that alter the microenvironment of chondrocytes and facilitate the release of MMPs and proteoglycans, resulting in the degradation of the extracellular matrix of chondrocytes. Meanwhile, the degraded extracellular matrix debris can act as a DAMPS to further stimulate the activation of M1 macrophages and then aggravate synovial inflammation, thus forming a vicious cycle that aggravates the progression of arthritis [42–44]. Samavedi et al. found that after coculture of chondrocytes and LPS-stimulated macrophages, the secretion of MMP-13, IL-1β, TNF-α, IL-6 and other inflammatory factors in chondrocytes was significantly increased in relation to chondrocyte monoculture; moreover, cocultured macrophages were confirmed to produce larger amounts of IL-1β than macrophage monoculture [45]. Therefore, exploring the paracrine roles of M1 macrophages and chondrocytes in the process of osteoarthritis is essential. Therefore, we cocultured normal chondrocytes or osteoarthritic chondrocytes with M0 or M1 macrophages *in vitro* to explore the effects of astaxanthin on chondrocytes and macrophages in a coculture environment.

In summary, the main purpose of the current study was to elucidate whether astaxanthin and Rspo2 are involved in chondrocyte degeneration and macrophage M1 polarization, to examine the related mechanisms under mono-culture conditions and to explore the effects of astaxanthin on chondrocytes and macrophages in the context of coculture. In addition, a destabilization of the medial meniscus (DMM)-induced OA model was used *in vivo* to address the roles of astaxanthin and Rspo2 in the development of osteoarthritis, with a view to detecting critical approaches for the treatment of osteoarthritis.

## RESULTS

### Macrophage polarization in the synovial tissue of osteoarthritis patients

**Figure 1:**
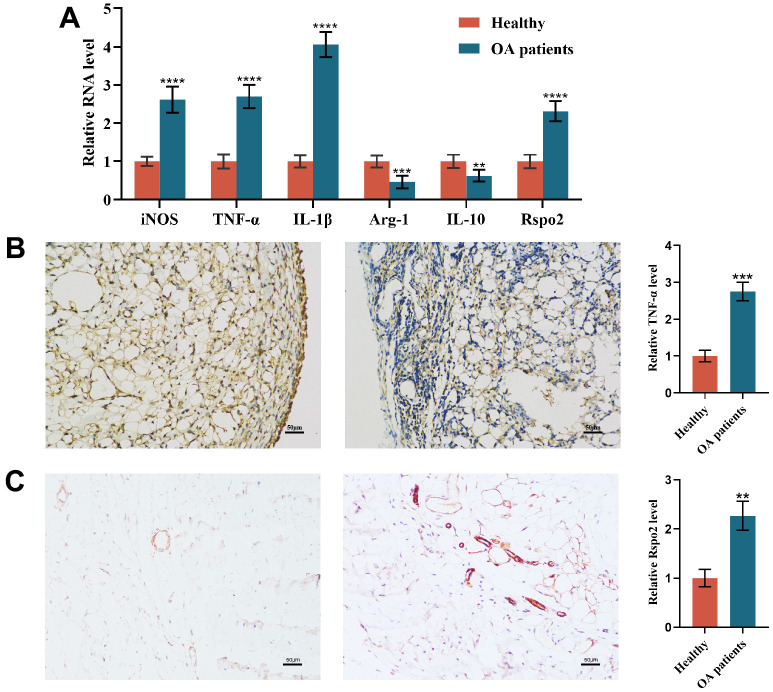
**Macrophage polarization in the synovial tissue of osteoarthritis patients.** (**A**) qRT-PCR was used to detect the gene expression of M1 macrophage markers (TNF-α, IL-1β, and iNOS), M2 macrophage markers (IL-10 and Arg-1) and Rspo2 in the synovial tissue of the two groups of patients. (**B**) TNF-α immunohistochemical staining for synovial inflammation in the two groups was performed. (**C**) Rspo2 immunohistochemical staining for synovial inflammation in the two groups was performed. The data are represented as the mean ± S.D. of three independent experiments (n = 3). ns: Not significant vs Normal group; **p < 0.01 vs Normal group; ***p < 0.001 vs Normal group; ****p < 0.0001 vs Normal group.

To investigate the role of macrophage polarization in osteoarthritis, we examined the expression of M1 and M2 macrophage markers in synovial tissue. We observed that the gene expression levels of M1 macrophage markers (TNF-α, IL-1β, and iNOS) and Rspo2 were significantly enhanced, while the gene expression levels of M2 macrophage markers (IL-10 and Arg-1) were significantly inhibited in synovial tissues of osteoarthritis patients relative to those in healthy controls. (Figure 1A). Immunohistochemistry showed that the expression of inflammatory protein (TNF-α) and Rspo2 was significantly higher than that of healthy people, and the nuclear morphology was changed significantly (Figure 1B, 1C).

### Effect of astaxanthin on the activity of chondrocytes and macrophages and the phenotypic assessment of NCs and OACs

**Figure 2:**
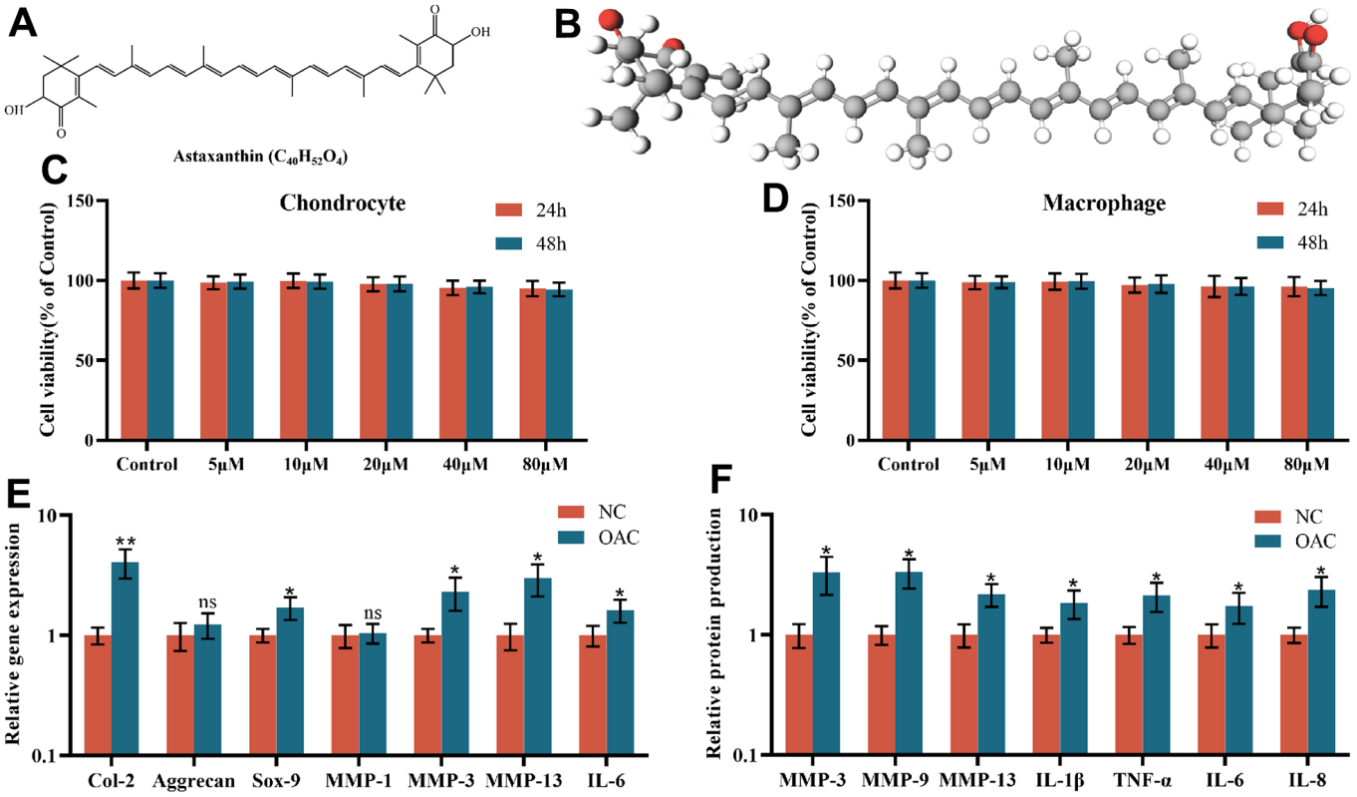
(**A**) The chemical structure of astaxanthin. (**B**) Three-dimensional (3D) structure of astaxanthin. (**C**) The cytotoxic effect of astaxanthin (5, 10, 20, 40, and 80 μM) exposure for 24 and 48 h on chondrocytes was determined using a CCK8 assay. (**D**) The cytotoxic effect of astaxanthin (5, 10, 20, 40, and 80 μM) exposure for 24 and 48 h on macrophages was determined using a CCK8 assay. (**E**) Relative gene expression of osteoarthritic chondrocytes (OACs) and normal chondrocytes (NCs). (**F**) Relative protein expression of osteoarthritic chondrocytes (OACs) and normal chondrocytes (NCs). The data are represented as the mean ± S.D. of three independent experiments (n = 3). ns: Not significant vs NC group; *p < 0.05 vs NC group; **p < 0.01 vs NC group.

The planar structure and three-dimensional (3D) structure of astaxanthin (Figure 2A, 2B). The cytotoxic effects of astaxanthin on chondrocytes and macrophages were examined at different concentrations (5, 10, 20, 40, and 80 μM) for 24 h or 48 h, and the results indicated that cell viability was not affected at these concentrations after 24 h or 48 h of exposure to astaxanthin (Figure 2C, 2D).

Although the gene expression levels of Aggrecan and MMP-1 were not significantly different between NCs and OACs, the gene expression levels of Col-2, Sox-9, MMP-3, MMP-13 and IL-6 were all elevated in OACs compared to NCs (Figure 2E). At the protein level, in relation to NCs, OACs produced significantly higher levels of MMP-3, MMP-9, MMP-13, IL-1β, TNF-α, IL-6 and IL-8 (Figure 2F), demonstrating significant inherent phenotypic differences between NCs and OACs.

### Effects of astaxanthin on normal chondrocytes (NCs) and osteoarthritic chondrocytes (OACs)

**Figure 3:**
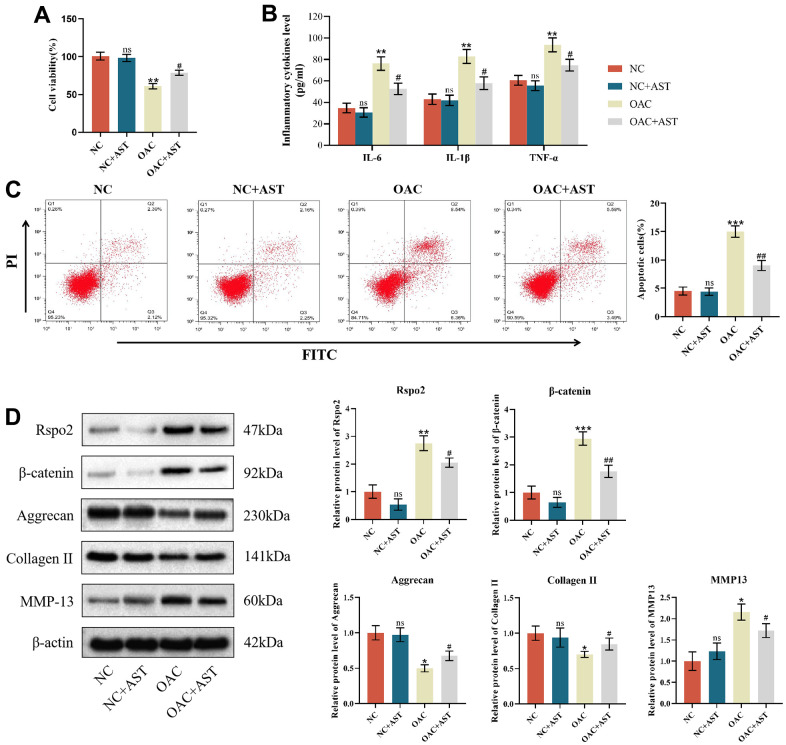
**Effects of astaxanthin on normal chondrocytes (NCs) and osteoarthritic chondrocytes (OACs).** (**A**) Effect of astaxanthin on the activity of NCs and OAC cells detected by CCK8. (**B**) Effect of astaxanthin on the levels of inflammatory factors (IL-6, IL-1β, and TNF-α) in NCs and OACs detected by ELISA. (**C**) Effect of astaxanthin on the apoptosis rate of NCs and OACs detected by flow cytometry with annexin V-FITC/PI analysis. (**D**) Western blot was used to detect the effect of astaxanthin on the expression of NC- and OAC-related proteins. The data are represented as the mean ± S.D. of three independent experiments (n = 3). ns: Not significant vs NC group; *p < 0.05 vs NC group; **p < 0.01 vs NC group; ***p < 0.001 vs NC group; ^#^*p* < 0.05 vs OAC group; ^##^*p* < 0.01 vs OAC group.

After pretreatment with astaxanthin (10 μmol/L) for 24 h, cell viability, apoptosis ratios, and protein expression of inflammatory factors, catabolic markers and anabolic markers were examined in NCs and OACs. We found that under conditions of astaxanthin, cell viability and protein expression of type II collagen and aggrecan were all elevated, while apoptotic cells and protein expression of IL-6, IL-1β, TNF-α, Rspo2, β-catenin and MMP-13 were all attenuated compared with those cultured alone in OACs. However, in NCs, cell viability, apoptosis ratios, and protein expression of inflammatory factors, catabolic markers and anabolic markers were not affected by exposure to astaxanthin (Figure 3A–3D).

### Astaxanthin abrogated the process of OA by inhibiting the Rspo2-related Wnt/β-catenin pathway in OACs

**Figure 4:**
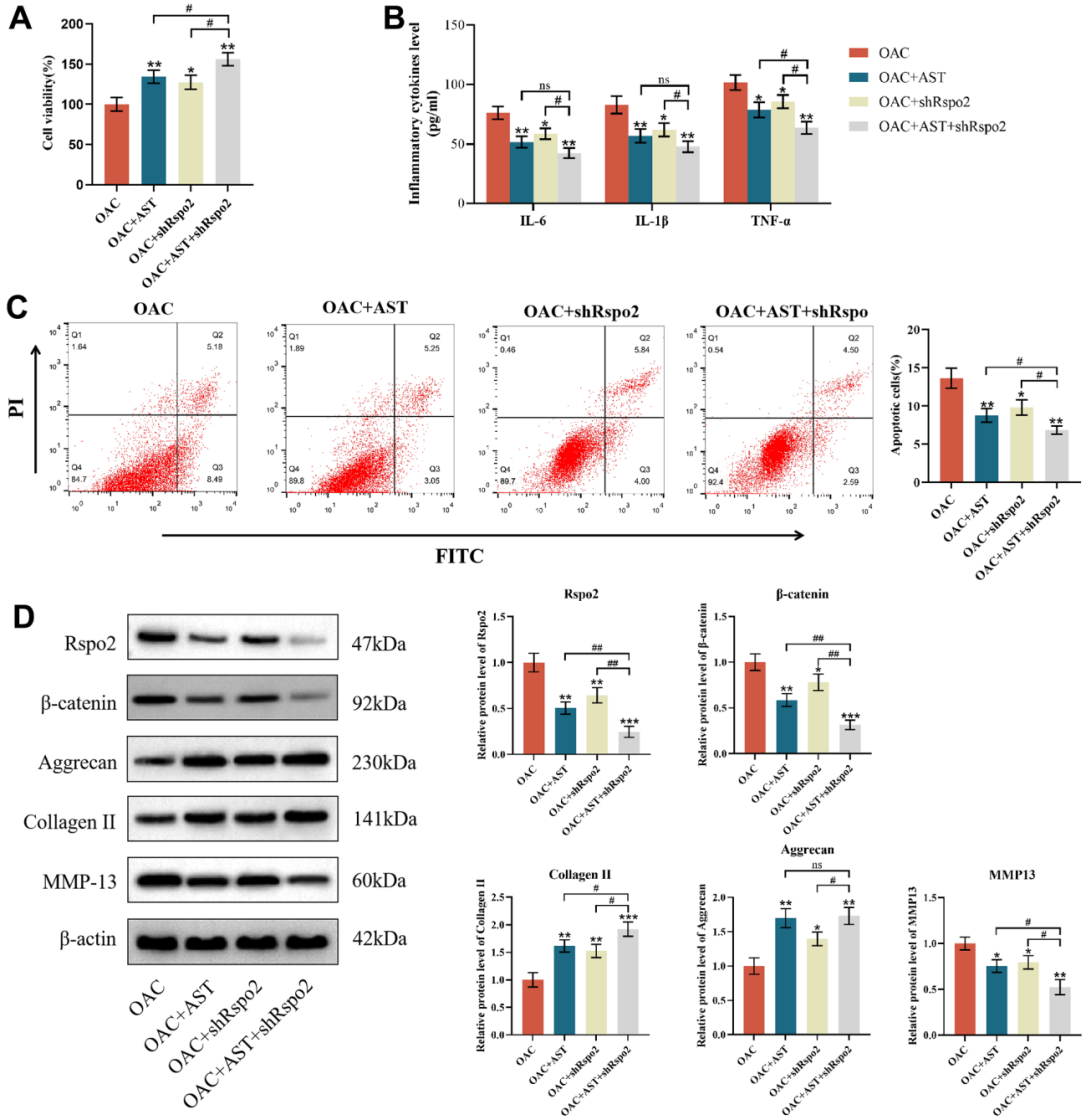
**Astaxanthin abrogated the process of OA by inhibiting the Rspo2-related Wnt/β-catenin pathway.** (**A**) Effect of astaxanthin, shRspo2 or astaxanthin combined with shRspo2 on the activity of OAC cells detected by CCK8. (**B**) Effect of astaxanthin, shRspo2 or astaxanthin combined with shRspo2 on the levels of inflammatory factors (IL-6, IL-1β, and TNF-α) in OACs detected by ELISA. (**C**) Effect of astaxanthin, shRspo2 or astaxanthin combined with shRspo2 on the apoptosis rate of OAC cells detected by flow cytometry with annexin V-FITC/PI analysis. (**D**) Western blotting was used to detect the effect of astaxanthin, shRspo2 or astaxanthin combined with shRspo2 on the expression of OAC-related proteins. The data are represented as the mean ± S.D. of three independent experiments (n = 3). *p < 0.05 vs the OAC group; **p < 0.01 vs the OAC group; ***p < 0.001 vs the OAC group; ns: Not significant vs the OAC+AST+shRspo2 group; ^#^*p* < 0.05 vs the OAC+AST+shRspo2 group; ^##^*p* < 0.01 vs the OAC+AST+shRspo2 group.

To determine whether Rspo2 and β-catenin are involved in astaxanthin-mediated prevention of OA deterioration, we pretreated OACs with shRNA targeting Rspo2, astaxanthin and both (Rspo2 shRNA+ astaxanthin) or untreated OACs as controls. We have detected that pre-treatment with astaxanthin or Rspo2 shRNA, compared to the control group, can enhance cell viability, increase protein expression of type II collagen and aggregating protein, and reduce cell apoptosis. It also decreases the expression of IL-6, IL-1β, TNF-α, Rspo2, β-catenin, and MMP-13. The combined use of astaxanthin and Rspo2 shRNA had more significant effects (Figure 4A–4D).

### Effects of astaxanthin on M0 macrophages (NM) and proinflammatory M1 macrophages (AM)

**Figure 5:**
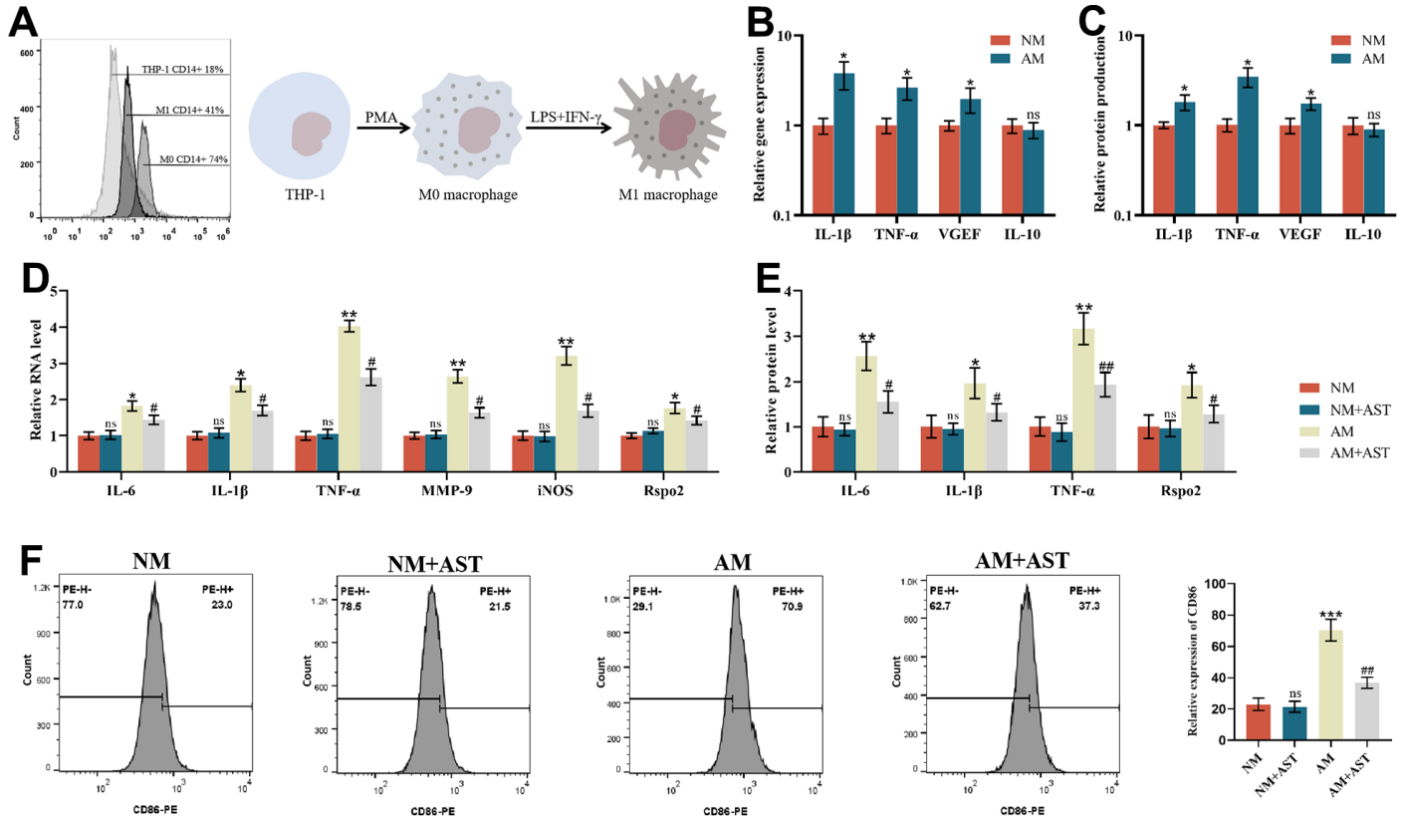
**Effects of astaxanthin on M0 macrophages (NM) and proinflammatory M1 macrophages (AM).** (**A**) Verification of the differentiation from THP-1 cells to resting M0 macrophages (NM) and proinflammatory M1 macrophages (AM) via flow cytometry analysis of CD14 cell surface expression. (**B**) The relative gene expression of M0 macrophages (NM) and proinflammatory M1 macrophages (AM) was measured by qRT-PCR. (**C**) The relative protein expression of M0 macrophages (NM) and proinflammatory M1 macrophages (AM) was detected by ELISA. (**D**) Effect of astaxanthin on M0 macrophages (NM) and proinflammatory M1 macrophages (AM) measured by qRT-PCR. (**E**) Effect of astaxanthin on M0 macrophages (NM) and proinflammatory M1 macrophages (AM) detected by ELISA. (**F**) Flow cytometry was used to detect the expression of CD86 (CD86 is a special surface phenotype marker of M1 macrophages).

PMA was used to stimulate the THP-1 monocytic cell line to differentiate into stationary M0 macrophages (rM0) to establish an inflammation model with a phenotype resembling that of human monocyte-derived macrophages (MDM), which were then differentiated into the M1 macrophage subtype [45, 46]. The differentiation was then verified by the expression of the M0 macrophage-specific surface marker CD14. Flow cytometry analysis showed that 18% of THP-1 cells were CD14 positive. After treatment with PMA, flow cytometry analysis showed that 76% of cells were CD14 positive, which proved that THP-1 cells had differentiated into resting M0 macrophages. After IFN-γ and LPS treatment, flow cytometry analysis showed that 41% of the cells were CD14 positive. A decrease in CD14 surface marker expression demonstrated that resting M0 macrophages were activated into proinflammatory M1 macrophages (Figure 5A). To further confirm this activation, we analyzed different parameters of M0 macrophages (NM) and proinflammatory M1 macrophages (AM). Although there were no significant differences in the gene expression of IL-10 between NM and AM, the gene expression levels of IL-1β, TNF-α, and VEGF were all elevated in AM compared to NM (Figure 5B). At the protein level, AM produced significantly higher levels of IL-1β, TNF-α, and VEGF compared to NM (Figure 5C). These results demonstrated significant inherent phenotypic differences between NM and AM. The results of qRT-PCR, ELISA, and flow cytometry showed that, compared with M0 macrophages (NM), the expression of IL-6, IL-1β, TNF-α, MMP-9, iNOS, Rspo2 and the proportion of M1 macrophages of M1 macrophages (AM) was increased significantly. After pretreatment with astaxanthin (10 μmol/L) for 24 h, the expression of the above indicators and the proportion of proinflammatory M1 macrophages (AM) was decreased significantly, while that of M0 macrophages (NM) did not change significantly (Figure 5D–5F).

### The effects of macrophage coculture on NCs

**Figure 6:**
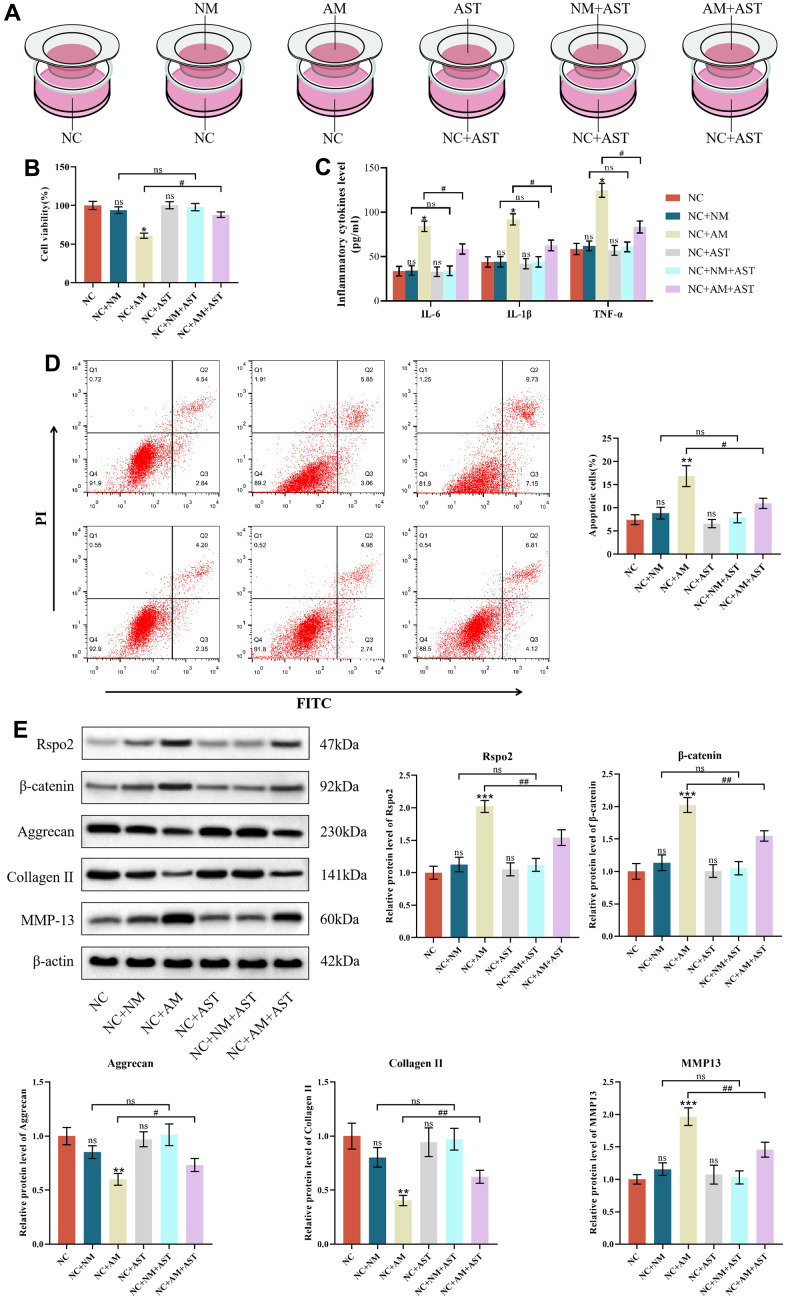
**The effects of macrophage coculture on NCs.** (**A**) NC indicates normal chondrocytes cultured alone, NC+NM indicates coculture of normal chondrocytes with M0 macrophages, NC+AM indicates coculture of normal chondrocytes with M1 macrophages, NC+AST indicates normal chondrocytes with astaxanthin, NC+NM+AST indicates coculture of normal chondrocytes with M0 macrophages with astaxanthin, and NC+AM+AST indicates coculture of normal chondrocytes with M1 macrophages with astaxanthin. (**B**) CCK8 was used to detect the activity of chondrocytes in each group. (**C**) The levels of chondrocyte inflammatory factors (IL-6, IL-1β, and TNF-α) in each group were detected by ELISA. (**D**) Apoptosis rate of chondrocytes in each group was detected by flow cytometry with annexin V-FITC/PI analysis. (**E**) Western blot was used to detect the expression level of chondrocyte-related proteins in each group. The data are represented as the mean ± S.D. of three independent experiments (n = 3). ns: Not significant; *p < 0.05 vs NC group; **p < 0.01 vs NC group; ***p < 0.001 vs NC group; ^#^*p* < 0.05 vs NC+AM group; ^##^*p* < 0.01 vs NC+AM group.

To explain the paracrine interaction of macrophages and chondrocytes, we first established a coculture system to explore the effects of macrophage coculture on the functions of NCs to mimic the early stage of OA. We determined that only coculture with AMs but not NMs, could elevate apoptotic cells and the protein expression of IL-6, IL-1β, TNF-α, Rspo2, β-catenin and MMP-13 and subsequently diminish cell viability and the protein expression of type II collagen and aggrecan compared to NCs in monoculture. Astaxanthin only exhibited therapeutic effects in the NCs+AMs groups, rather than in the NCs groups or NCs+NMs groups (Figure 6A–6E).

### The roles of macrophage coculture in OACs

**Figure 7:**
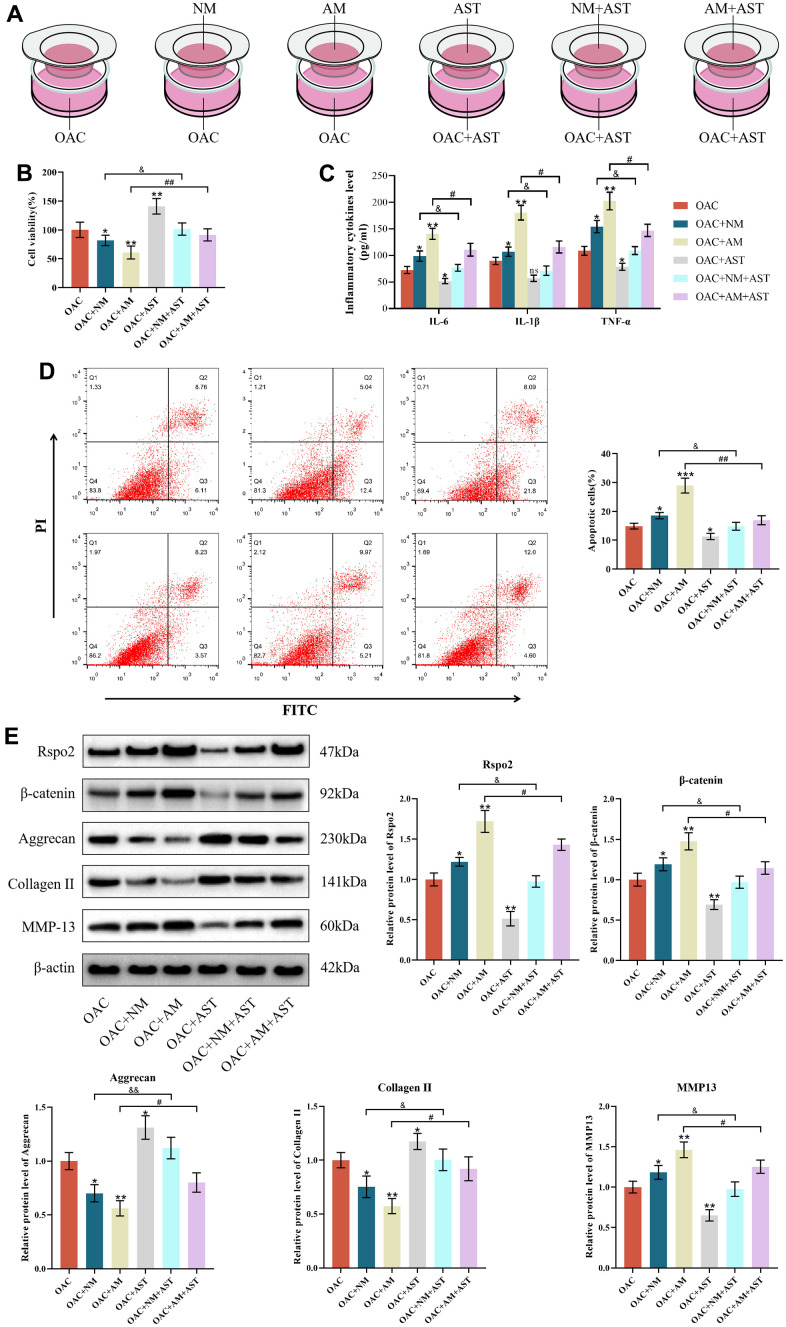
**The roles of macrophage coculture in OACs.** (**A**) OAC indicates osteoarthritic chondrocytes cultured alone, OAC+NM indicates coculture of osteoarthritic chondrocytes with M0 macrophages, OAC+AM indicates coculture of osteoarthritic chondrocytes with M1 macrophages, OAC+AST indicates osteoarthritic chondrocytes with astaxanthin, OAC+NM+AST indicates coculture of osteoarthritic chondrocytes with M0 macrophages with astaxanthin, and OAC+AM+AST indicates coculture of osteoarthritic chondrocytes with M1 macrophages with astaxanthin. (**B**) CCK8 was used to detect the activity of chondrocytes in each group. (**C**) The levels of chondrocyte inflammatory factors (IL-6, IL-1β, and TNF-α) in each group were detected by ELISA. (**D**) Apoptosis rate of chondrocytes in each group was detected by flow cytometry with annexin V-FITC/PI analysis. (**E**) Western blot was used to detect the expression level of chondrocyte-related proteins in each group. The data are represented as the mean ± S.D. of three independent experiments (n = 3). ns: Not significant; *p < 0.05 vs the OAC group; **p < 0.01 vs the OAC group; ***p < 0.001 vs the OAC group; ^&^*p* < 0.05 vs the OAC+NM group; ^&&^*p* < 0.01 vs the OAC+NM group; ^#^*p* < 0.05 vs the NC+AM group; ^##^*p* < 0.01 vs the NC+AM group.

We further evaluated the roles of macrophage coculture in the functions of OACs to mimic the progressive stage of OA. Our results revealed that coculture with both NMs and AMs resulted in enhanced apoptotic cells and protein expression of IL-6, IL-1β, TNF-α, Rspo2, β-catenin and MMP-13, accompanied by attenuated cell viability and protein expression of type II collagen and aggrecan in relation to OACs in monoculture. The effects were more pronounced when cocultured with AMs than when cocultured with NMs. Astaxanthin reversed these changes among all three groups (NCs groups, NCs+NMs groups, and NCs+AMs groups). (Figure 7A–7E).

### The effects of chondrocyte coculture on AMs

**Figure 8:**
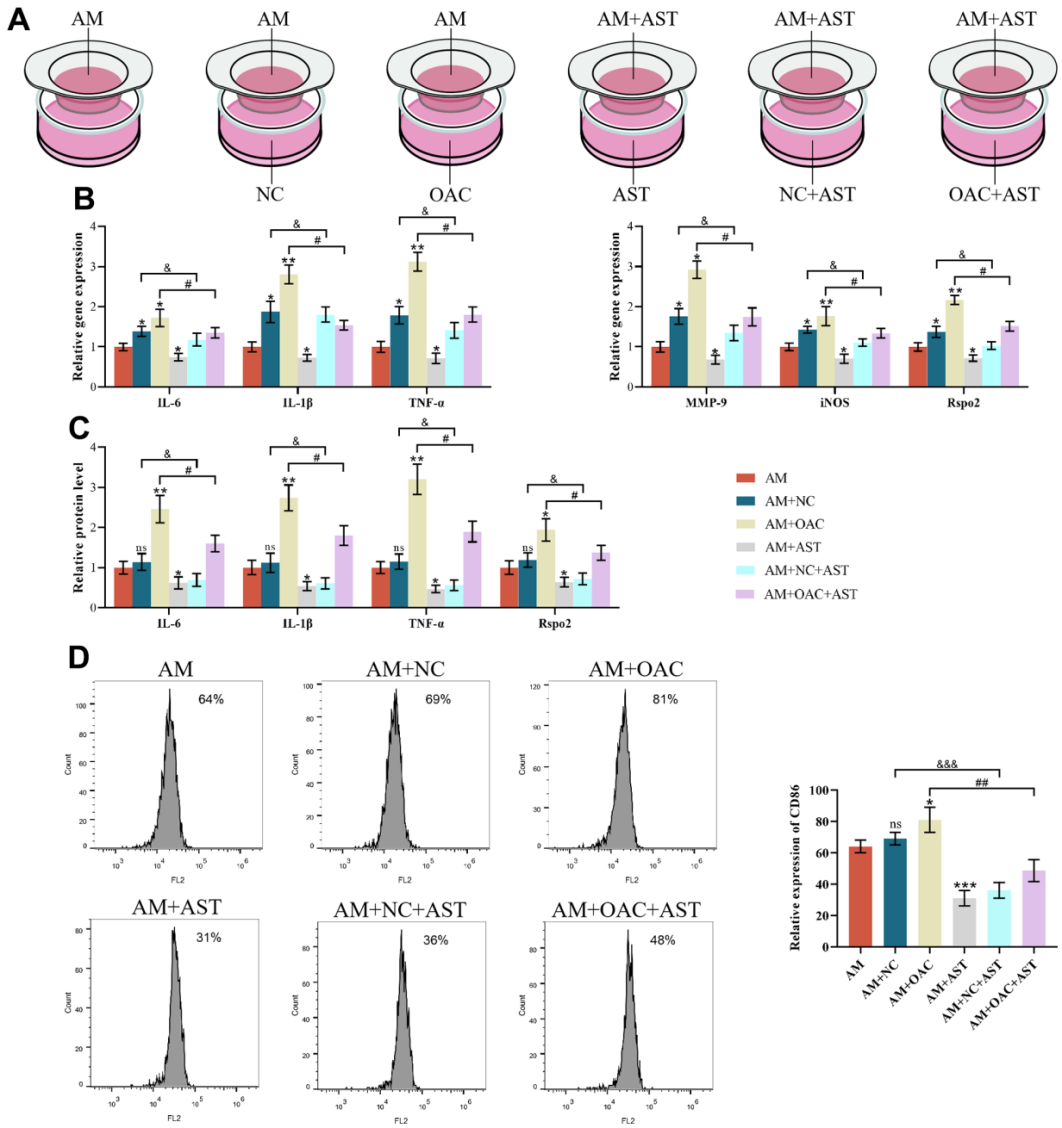
**The effects of chondrocyte coculture on AMs.** (**A**) AM indicates M1 macrophages cultured alone, AM+NC indicates coculture of M1 macrophages with normal chondrocytes (NC), AM+OAC indicates coculture of M1 macrophages with osteoarthritic chondrocytes, AM+AST indicates M1 macrophages with astaxanthin, AM+NC+AST indicates coculture of M1 macrophages with normal chondrocytes (NC) with astaxanthin, and OAC+AM+AST indicates coculture of osteoarthritic chondrocytes with M1 macrophages with astaxanthin. (**B**) qRT-PCR was used to detect the expression of IL-6, IL-1β, TNF-α, MMP-9, iNOS, and Rspo2 in macrophages. (**C**) ELISA was used to detect the expression levels of IL-6, IL-1β, TNF-α, and Rspo2 in macrophages of each group. (**D**) Flow cytometry was used to detect the expression of CD86 (CD86 is a special surface phenotype marker of M1 macrophages). The data are represented as the mean ± S.D. of three independent experiments (n = 3). ns: Not significant; *p < 0.05 vs AM group; **p < 0.01 vs AM group; ***p < 0.001 vs AM group; ^&^*p* < 0.05 vs AM+NC group; ^&&&^*p* < 0.001 vs AM+NC group; ^#^*p* < 0.05 vs AM+OAC group; ^##^*p* < 0.01 vs AM+OAC group.

In addition, we aimed to explore the crosstalk between macrophages and chondrocytes. We found that when cocultured with NCs, the gene expression levels of IL-6, IL-1β, TNF-α, MMP-9, iNOS and Rspo2 were markedly increased, while the proportion of M1 macrophages, protein expression levels of inflammatory factors (IL-6, IL-1β, TNF-α) and Rspo2 were not affected compared with AM mono-culture. Astaxanthin blocked the elevated gene expression levels of IL-6, IL-1β, TNF-α, MMP-9, iNOS and Rspo2. However, upon OAC culture, the proportion of M1 macrophages, gene expression levels of IL-6, IL-1β, TNF-α, MMP-9, iNOS, and Rspo2 and protein expression levels of IL-6, IL-1β, TNF-α, and Rspo2 were all enhanced relative to AM mono-culture. Astaxanthin rescued all the changes (Figure 8A–8D).

### Astaxanthin inhibited the development of OA by blocking Rspo2 in an *in vivo* DMM model

**Figure 9:**
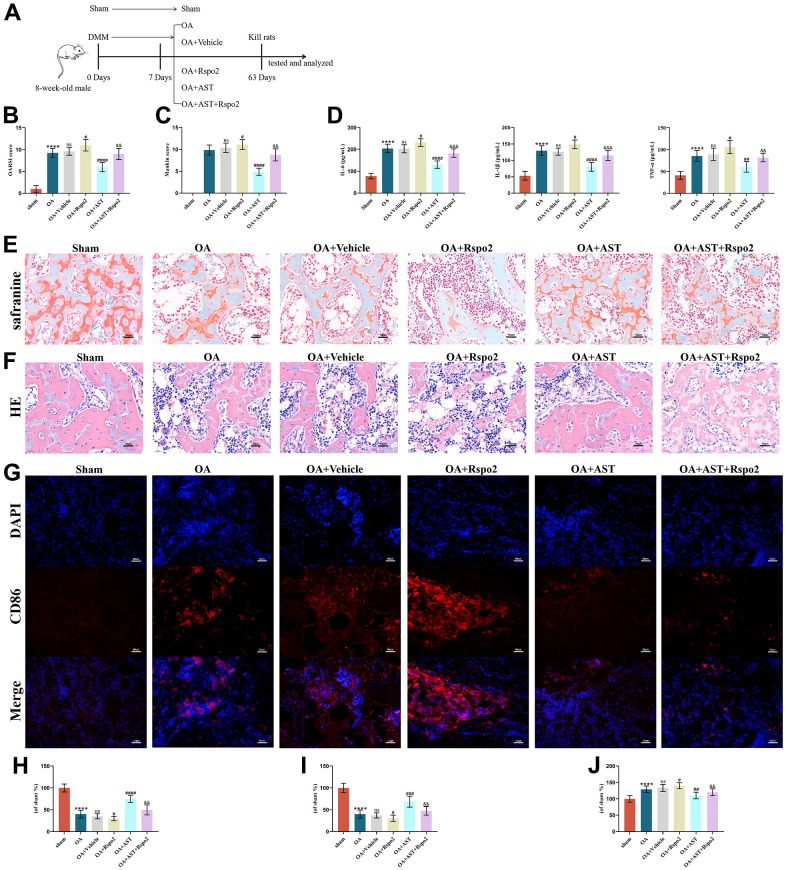
**Astaxanthin inhibited the development of OA by blocking Rspo2 in an *in vivo* DMM model.** (**A**) A graphic scheme of OA induction and AST administration. (**B**) The joint lesions were evaluated by the OARSI scoring system. (**C**) The joint lesions were evaluated by the Mankin scoring system. (**D**) ELISA was used to detect the expression of IL-6, IL-1β, and TNF-α in joint cavity effusion. (**E**, **H**) The cartilage tissues were analyzed via Safranin O staining. (**F**, **I**) The cartilage tissues were analyzed via HE staining. (**G**, **J**) Immunofluorescence staining for CD86. The data are represented as the mean ± S.D. of three independent experiments (n = 8). ns: Not significant; ****p < 0.0001 vs Sham group; ^#^*p* < 0.05 vs OA group; ^##^*p* < 0.01 vs OA group; ^###^*p* < 0.001 vs OA group; ^####^*p* < 0.0001 vs OA group; ^&&^*p* < 0.01 vs the OA+Rspo2 group; ^&&&^*p* < 0.001 vs the OA+Rspo2 group.

To investigate the effects of astaxanthin and Rspo2 on the onset and process of OA *in vivo*, we established a surgically induced DMM rat OA model. Intra-articular injection of Rspo2 in the OA group resulted in significantly higher OARSI scores and Mankin scores, while Astaxanthin markedly decreased OARSI scores and Mankin scores in OA rats. We examined the inflammatory factors in the synovial fluid by ELISA and cartilage quality by staining with HE and Safranin-O in different groups, and examined the proportion of M1 macrophages by immunofluorescence. We confirmed that the protein expression levels of IL-6, IL-1β, and TNF-α were all increased in the OA groups compared with the sham groups. The levels of these inflammatory factors were significantly elevated after intra-articular injection of Rspo2 in the OA groups. Astaxanthin prevented the secretion of IL-6, IL-1β, and TNF-α in both the OA and OA+Rspo2 groups. HE staining and Safranin O staining in the OA groups showed weaker staining than that in the sham groups, while the OA+Rspo2 groups exhibited the weakest staining. Pretreatment with astaxanthin resulted in stronger staining both in the OA groups and in the OA+Rspo2 groups. The proportion of M1 macrophages in the OA groups is higher than that in sham groups, while the OA+Rspo2 groups has the highest proportion of M1 macrophages. Pretreatment with astaxanthin reduced the proportion of M1 macrophages both in the OA groups and in the OA+Rspo2 groups. (Figure 9A–9G).

## DISCUSSION

**Figure 10:**
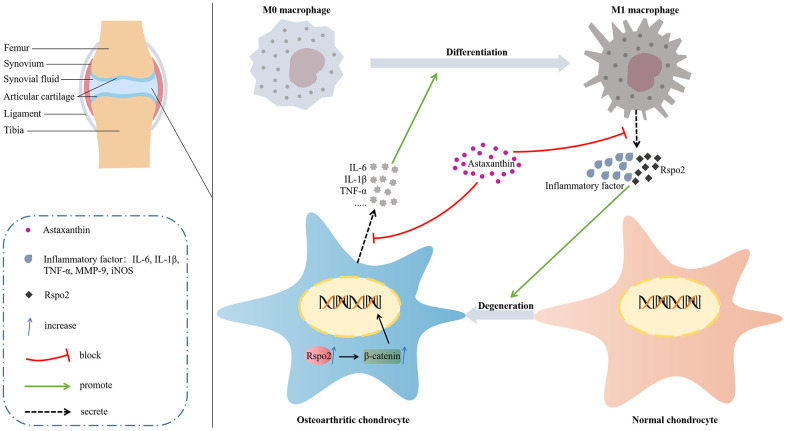
Molecular mechanism diagram.

Degeneration of chondrocytes, degradation of ECM and activation of synovial M1 macrophages play indispensable roles in the onset and aggravation of osteoarthritis; therefore, exploring efficient approaches that could maintain the expression of ECM markers in chondrocytes and prevent the secretion of inflammatory cytokines by M1 macrophages appears central to the treatment of osteoarthritis. Here, we established that Rspo2 was a critical determinant in the onset and aggravation of OA based on the fact that in *in vitro* cultures, Rspo2 exhibited its specific functions by acting as one of inflammatory factors in M1 macrophages, elevating catabolic markers and blocking anabolic markers in chondrocytes. In an *in vivo* DMM-induced OA model, Rspo2 was shown to promote the development of OA. Astaxanthin was first established for reversing Rspo2-stimulated adverse effects both *in vitro* and *in vivo* in the progression of OA (Figure 10).

Despite the complexity of the pathogenesis and course of osteoarthritis, exploring the critical determinants appears vital in the treatment of osteoarthritis. We observed that in addition to generally viewed inflammatory factors iNOS, TNF-α and IL-1β, Rspo2 in the synovial tissues from patients who underwent arthroplasty were significantly enhanced, while the gene expression of IL-10 and Arg-1 was downregulated in relation to healthy controls. Our results revealed that Rspo2 may be a pivotal factor in the development of synovial inflammation related to the progression of OA. Our results were in agreement with the study from Okura, which confirmed elevated Rspo2 protein expression in synovial fluid from OA patients [35]. An earlier study by Zhang also revealed that as an important protein secreted by M1 macrophages, Rspo2 exacerbates the development of experimental osteoarthritis, implicated its potential role in OA treatment [13]. Rspo2 was also proved to play markedly roles in pathological crosstalk among macrophages, chondrocytes and synovial fibroblasts in a post-traumatic osteoarthritis setting [47]. As Rspo2 caught our attention, we further assessed the function of Rspo2 in macrophages cultured *in vitro*. We first explored that in addition to the widely accepted elevation of the gene expression of IL-6, IL-1β, TNF-α, MMP-9, and iNOS and the protein expression of IL-6, IL-1β, and TNF-α, the gene and protein levels of Rspo2 were both enhanced in AMs compared to NMs. Our results indicated that Rspo2 appear vital in M1 macrophage polarisation.

Astaxanthin, a unique carotenoid, exerts various functions, including antiapoptotic, antioxidative damage, and anti-inflammatory effects. Massive evidence has confirmed that astaxanthin can be used in various diseases, including neurodegenerative disorders, diabetes, and gastrointestinal disease. Astaxanthin mixture have already been confirmed to alleviate the symptom and process of osteoarthritis in both animal models and OA adults [27, 28]. Intra-articular injection of Rspo2 was reported to be a good alternative in the treatment of OA instead of corticosteroid and hyaluronic acid [48]. Several researchers have demonstrated that astaxanthin exhibits anti-inflammatory effects by suppressing inflammatory cytokines secreted by macrophages cultured *in vitro* [30, 31]. For example, astaxanthin was also observed to suppress neuroinflammation by regulating microglia M1 activation [49]. Thus, we speculated the involvement of astaxanthin in the regulation of M1 macrophage polarisation in our current setting. We found that astaxanthin could abrogate the function of AMs by blocking the gene expression of Rspo2, IL-6, IL-1β, TNF-α, MMP-9, and iNOS and the protein expression of Rspo2, IL-6, IL-1β, and TNF-α in 2D cultures. Interestingly, astaxanthin had no role in NMs in 2D cultures. To the best of our knowledge, our results are the first to reveal the roles of astaxanthin as an upstream regulator in the suppression of Rspo2-related inflammatory effects in AMs.

How to maintain the functions of chondrocytes under pathological conditions has been widely proven to be critical in the treatment of osteoarthritis. The optimized chondrocytes exhibited a lower apoptosis rate, less secretion of inflammatory factors, upregulated anabolic markers and downregulated catabolic markers of the ECM. IL-6, IL-1β, and TNF-α are the most important inflammatory factors, while MMP-13 is widely used as a pivotal catabolic marker, and aggrecan and COL2 are commonly considered to be two main anabolic markers. As Rspo2 and astaxanthin are two key mediators in the regulation of AMs, we then sought to explore the effects of Rspo2 and astaxanthin on the metabolism of chondrocytes. A search of the literature revealed few studies about the roles of Rspo2 and astaxanthin in chondrocytes. Rspo2 is widely accepted as an upstream regulator of the Wnt/β-catenin pathway, and increasing evidence supports the involvement of the Wnt/β-catenin pathway in chondrocytes in the development of OA [39, 50–52]. Work from Zhi reported that Rspo2 was essential for the protein expression of IL-6 and TNF-α in IL-1β-treated chondrocytes [53]. In an earlier investigation, Chen and coworkers demonstrated that in chondrocytes, IL-1β could elevate the gene and protein expression of MMP-13, while astaxanthin reversed the change [54]. In two recent studies, IL-1β was reported to promote protein expression of MMP-13 and abolish protein expression of COL2 in chondrocytes, while astaxanthin had a rescuing effect [55, 56]. The former studies all used an IL-1β-induced *in vitro* osteoarthritic model and only examined a few inflammatory mediators. We first examined different states of chondrocytes, NCs and OACs, and more extensively their functions. We observed that the apoptosis rate, protein expression of Rspo2, β-catenin, IL-6, IL-1β, TNF-α, and MMP-13 were increased, and protein expression of aggrecan and COL2 were reduced in OACs in relation to NCs. In addition, we detected that only in OACs, other than in NCs, the application of astaxanthin attenuated the apoptosis rate, protein expression of Rspo2, β-catenin, IL-6, IL-1β, TNF-α, and MMP-13, and subsequently elevated the protein expression of aggrecan and COL2. Furthermore, in OACs, Rspo2 inhibition by its targeted shRNA significantly abolished the apoptosis rate and protein expression of IL-6, IL-1β, TNF-α, and MMP-13 and subsequently elevated the protein expression of aggrecan and COL2. Our results strongly revealed that astaxanthin seems to play noticeable roles in maintaining the functions of chondrocytes by blocking the Rspo2-related Wnt/β-catenin pathway under pathological conditions.

To accurately mimic the articular environment, a coculture model of chondrocytes and macrophages was used to detect the crosstalk between macrophages and chondrocytes. Several researchers have focused on the paracrine interaction of macrophages and chondrocytes in the inflammatory environment. NC and AM cocultures were used to mimic the early stage of OA, while OACs and AMs were used to mimic the progressive stage of OA. We selected NCs and NMs, NCs and AMs, OACs and NMs, and OACs and AMs in the coculture system to detect the effects of macrophage coculture on the functions of chondrocytes in relation to chondrocytes cultured alone. Our results revealed that in NCs, only coculture with AMs, not NMs, could aggravate the apoptosis and secretion of IL-6, IL-1β, TNF-α, MMP-13, Rspo2, and β-catenin and inhibit the protein expression of aggrecan and COL2 compared with culture alone, while astaxanthin exhibited therapeutic effects by suppressing those inflammatory factors and elevating the ECM markers aggrecan and COL2 in the coculture system. Our results regarding aggrecan, COL2, IL-6, IL-1β, TNF-α and MMP-13 are consistent with data from Samavedi and collaborators [45]. Work from Hoff and coworkers also demonstrated that the amounts of human chondrocytes decreased and IL-6 levels were elevated in synovial fluids from OA patients [57]. Unexpectedly, in OACs, either coculture with AMs or NMs exacerbated the apoptosis and secretion of IL-6, IL-1β, TNF-α, MMP-13, Rspo2, and β-catenin and inhibited the protein expression of aggrecan and COL2 in relation to culture alone, and the effects were significantly stronger under AM conditions. Among all three groups, IL-6, IL-1β, TNF-α, MMP-13, Rspo2, and β-catenin secretion was prevented in combination with enhanced protein expression of aggrecan and COL2 in response to astaxanthin. Our data are similar to those of the majority of *in vitro* OAC and AM coculture systems. For example, an earlier study by Duan revealed that in human primary osteoarthritis chondrocytes, coculture with LPS-pretreated THP-1 cells could promote the apoptosis and secretion of inflammatory factors and prevent the proliferation of chondrocytes compared with chondrocyte monoculture [58]. However, Samavedi observed that in comparison to the OAC monoculture control, the protein expression levels of IL-6, IL-1β, TNF-α, and MMP-13 were significantly enhanced, while aggrecan and COL2 were not changed upon AM coculture [45]. The discrepancy in the results may be attributable to variations in stimuli environment, stimuli time and settings. In an OAC and NM coculture system, researchers established that IL-1β elevated IL-6 expression in chondrocyte monoculture but had no effects on M0 macrophage monoculture. In contrast, IL-6 expression was noticeably elevated in the OAC and NM coculture system, and resveratrol had protective effects by inhibiting the paracrine interaction between OACs and NMs [59]. The trend of IL-6 is consistent with data in our current study, perhaps due to the inflammatory amplification loop in the OAC and NM coculture system. The lower apoptosis rate, lower secretion of proinflammatory factors and higher expression of anabolic factors in the OAC and NM coculture system than in the OAC and AM coculture system suggested the potential roles of anti-inflammatory regulators in the alleviation of OA. In addition, we selected AMs and NCs and AMs and OACs in the coculture system to detect the roles of chondrocyte coculture in the functions of macrophages compared with macrophages cultured alone. We observed that in AMs, coculture with NCs resulted in a slight elevation of IL-6, IL-1β, TNF-α, MMP-9, iNOS, and Rspo2 gene expression, while IL-6, IL-1β, TNF-α, and Rspo2 protein expression was not affected compared to culture alone, but when cocultured with OACs, these inflammatory mediators were noticeably enhanced at both the gene and protein levels. Astaxanthin reversed these changes in all three groups. Our results are in part consistent with an earlier investigation that reported that IL-1β gene expression increased in AM and NC coculture, and both IL-1β gene and protein expression were enhanced in AM and OAC coculture. Furthermore, VEGF-A or Arginase-1 gene levels associated with an M2 phenotype were also elevated in that study [45]. Our data clearly showed that in the early stage of OA, AM and NC coculture promoted M1 phenotype-related proinflammatory factor expression at the gene level. In the progressive stage of OA, AM and OAC coculture promoted M1 phenotype-related proinflammatory factor expression at both the gene and protein levels and did not exclude a possible role for the involvement of M2 phenotype genes and proteins. To the best of our knowledge, this is the first study to analyze the function of astaxanthin and Rspo2 in a chondrocyte and macrophage coculture system at different stages.

We further focused on the effects of astaxanthin and Rspo2 on the development of OA in an *in vivo* DMM-induced OA model. HE staining and Safranin O staining revealed that in relation to the sham group, cartilage damage was promoted in the DMM-induced OA group, and the quality of cartilage worsened upon intra-articular injection of Rspo2, while astaxanthin had protective effects on the destruction of cartilage in those groups. In addition to the negative effects of Rspo2 and the positive effects of astaxanthin *in vitro*, our data strongly suggested that Rspo2 was also proved to worsen the development of OA, whereas astaxanthin reduced the advancement *in vivo*.

Although the current study clarifies for the first time that astaxanthin and Rspo2 are two critical determinants in the onset and progression of OA, some deficiencies remain. For instance, in our work, we solely considered the role of M1 macrophages, despite the fact that other studies claimed that M2 macrophages were equally crucial for the control of OA. In addition, research is still needed to determine the roles of various other cell types, such as synovial fibroblasts and osteoblasts, in the onset of OA, apart from macrophages and chondrocytes. Furthermore, endoplasmic reticulum, mitochondrial dysfunction, oxidative stress was also proved to be critical factors of OA pathology [53, 55]. In future, we would explore the more precise mechanisms of astaxanthin and Rspo2 in the process of OA, focusing on their roles in endoplasmic reticulum, mitochondrial dysfunction and oxidative stress.

## CONCLUSIONS

Above all, we determined that astaxanthin blocked the process of OA by abolishing the secretion of Rspo2 and other related inflammatory factors in AMs, as well as promoting the synthesis of anabolic markers, diminishing the synthesis of catabolic markers and inflammatory factors by preventing the Rspo2-related Wnt/β-catenin pathway in chondrocytes.

## MATERIALS AND METHODS

### Isolation, culture, and treatment of chondrocytes

Human articular cartilage and synovial tissue from 6 cases of osteoarthritis treated with total knee arthroplasty and 6 cases of knee joint trauma requiring amputation without osteoarthritis were received from the Affiliated Changzhou No.2 People’s Hospital with Nanjing Medical University. Informed consent was obtained in all cases, and the study was approved by the Committee of Clinical Investigation of the Affiliated Changzhou Second People’s Hospital of Nanjing Medical University (2021-KY203-01). Synovial tissue from the suprapatellar bursa and tibial articular cartilage were collected during total knee arthroplasty from patients meeting diagnostic criteria for primary symptomatic knee OA defined by the American College of Rheumatology criteria [60], together with Kellgren Lawrence (KL) radiographic severity grade III or IV [61].

The tissue was digested with 0.25% trypsin (Gibco, USA) at 37° C for 30 minutes, followed by 0.2% type II collagenase at 37° C for 5 h. Cells were then passed through a 70-μm cell strainer and washed 3 times with sterile phosphate-buffered saline (PBS). The primary chondrocytes were cultured in high glucose DMEM (Gibco, USA) with 10% fetal bovine serum (FBS, Gibco, USA) and 1% penicillin-streptomycin antibiotics (Sigma-Aldrich, USA) at 37° C in a humidified atmosphere containing 5% CO_2_. The cell culture medium was changed every 2 to 3 days. Subsequent experiments used only primary or passaged one generation of chondrocytes.

Astaxanthin (Sigma-Aldrich, USA) was first dissolved in dimethyl sulfoxide (DMSO) and then diluted with complete medium to the desired concentration, with a final DMSO concentration of <0.1%. According to a literature report, 10 μM astaxanthin was chosen for subsequent experiments [56].

To prepare shRNA-expressing lentiviral vectors, double-stranded shRNA oligonucleotides (Sangon Biotech, China) were synthesized and cloned into osteoarthritic chondrocytes. shRspo2 (shR2): 5’-GATCCCGCTTGAAATTAATCCTAAATTCAAGAGATTTAGGATTAATTTCAAGCTTTTTGGAAA-3’ and 5’-AGCTTTTCCAAAAAGCTTGAAATTAATCCTAAATCTCTTGAATTTAGGATTAATTTCAAGCGG-3’.

### Differentiation of THP-1 monocytes into macrophages

To establish an inflammatory model, THP-1 monocytic cells (ATCC, USA) were seeded into cell culture flasks. By adding 100 nM phorbol-12-myristate 13-acetate (Sigma-Aldrich, Germany), THP-1 monocytic cells were differentiated into macrophages for 3 days. Then, the PMA-containing medium was removed. The differentiation of PMA-treated THP-1 cells was extended by incubating the cells in fresh culture medium for an additional 5 days. Cells that continued to receive regular growth medium were designated as nonactivated macrophages (NM).

### Differentiation of M0 macrophages into M1 macrophages

After the above differentiation (3 days PMA, 5 days resting), M0 macrophages (rM0) were activated to the M1 macrophages by adding 20 ng/ml IFN-γ (Sigma-Aldrich, Germany) and 500 ng/ml LPS (Sigma-Aldrich, Germany) to the fresh culture medium for 2 days. Cells activated with LPS and IFN-γ were designated as activated macrophages (AM).

### Cocultivation of chondrocytes [normal chondrocytes (NCs) or osteoarthritic chondrocytes (OACs)] and macrophages [M0 macrophages (NMs) or proinflammatory M1 macrophages (AMs)]

In this experiment, a Transwell chamber (Corning, USA) was used to establish a cell coculture system. Normal chondrocytes (NC) or osteoarthritic chondrocytes (OAC) were inoculated into the lower culture chamber of the 6-well Transwell chamber for culture. THP-1 cells were inoculated on a 6-well plate in the lower culture chamber (separate from 6-well Transwell plate) and then induced into M0 macrophages (NM) or proinflammatory M1 macrophages (AM) according to the above method. Finally, the Transwell upper culture chamber was transferred back to the 6-well Transwell plate covered with normal chondrocytes (NC) or osteoarthritis chondrocytes (OAC).

### Cell counting kit-8 (CCK-8)

According to the manufacturer’s protocol, the effect of astaxanthin on cell viability and the role of macrophages on chondrocyte viability were determined by CCK-8 assay. Chondrocytes or macrophages were seeded in 96-well plates and treated with different concentrations of astaxanthin (5, 10, 20, 40, and 80 μM) for 24 h and 48 h. Then, 100 μl of 10% CCK-8 reagent (Beyotime, China) was added to each well and incubated for 2 h, and the optical density (OD) at 450 nm of each well was measured with a microplate reader (Thermo Fisher Scientific, MA, USA).

After 7 days of coculture of macrophages and chondrocytes, CCK-8 reagent (Beyotime, China) was added to the Transwell chamber and incubated for 2 h. The cell supernatant was transferred from the Transwell chamber to a 96-well plate, and the optical density (OD) at 450 nm of each well was measured with a microplate reader (Thermo Fisher Scientific, MA, USA).

### Enzyme-linked immunosorbent assay (ELISA)

To detect the level of cytokines, ELISA was carried out using ELISA kits (MultiSciences and Gelatins, China) according to the manufacturer’s protocol. Cell supernatant was collected from chondrocytes or macrophages and centrifuged at 300 × g for 10 minutes. After killing the rats, the serum was collected, and the blood samples were agglutinated for 30 minutes and then centrifuged at 1,000 × g for 10 minutes. Then, the concentrations of IL-6, IL-1β, TNF-α, VEGF, IL-10, and Rspo2 were measured with ELISA kits. The absorbance at 450 nm was measured.

### Quantitative reverse transcription polymerase chain reaction (qRT-PCR)

The mRNA expression levels of IL-6, IL-1β, IL-10, iNOS, TNF-α, Arg-1, Rspo2, Col-2, Aggrecan, Sox-9, MMP-1, MMP-3, MMP-9, MMP-13, and VGEF were determined by qRT-PCR. TRIzol reagent (Beyotime, China) was used to isolate total RNA from tissues or cells. Then, Beyotime's first strand cDNA synthesis kit was used to reverse transcribe it into cDNA. Then, according to the instructions, SYBR Green qPCR mixture (Beyotime, China) was used for qRT-PCR. The reaction cycle conditions were as follows: 95° C for 2 minutes and 95° C for 15 seconds under predenaturation conditions; 40 cycles were conducted at 95° C for 15 seconds, 60° C for 20 seconds and 72° C for 30 seconds. The relative expression of each target gene was quantified and normalized by the 2^-Δ(ΔCt)^ method (ΔCt = Ct (sample - Ct (GAPDH), Δ(ΔCt) = ΔCt(sample) - ΔCt(control))) and GAPDH levels. The primer sequences (Sangon Biotech, China) used in qPCR are listed in Supplementary Table 1.

### Western blot

Chondrocytes were subjected to various treatments. Chondrocytes were rinsed three times with PBS and lysed with RIPA lysis buffer (Beyotime, China) supplemented with 1% phenylmethylsulfonyl fluoride (PMSF, Beyotime, China) and 1% deacetylase inhibitor cocktail (Beyotime, China) on ice for 30 minutes for western blot analysis. The cell lysates were ultrasonicated and centrifuged at 12,000 rpm for 15 minutes at 4° C in a precooled centrifuge. The supernatant was then harvested, and the protein concentration was quantified using a BCA assay kit (Beyotime, China). The proteins were then diluted using 5× sodium dodecyl sulfate-polyacrylamide gel electrophoresis (SDS-PAGE) loading buffer (Beyotime, China) and boiled at 100° C for 10 minutes. Equal amounts of the extracted proteins were separated using SDS-PAGE and transferred onto polyvinylidene fluoride (PVDF, Beyotime, China) membranes. The membranes were blocked with 5% skim milk for 1 hour at room temperature before being incubated with specific primary antibodies at 4° C overnight. The following day, the membranes were washed with TBST 3 times and incubated with the corresponding HRP-conjugated secondary antibodies (Beyotime, China) for 1 hour at room temperature. ECL chemiluminescence solution (Beyotime, China) was added for development, and the protein bands were exposed and photographed by a ChemiDocTM XRS System (Bio-Rad, USA). The gray values of the protein bands were analyzed by ImageJ software.

### Flow cytometry

The surface markers of stimulated cells were detected by flow cytometry. THP-1 cells, resting M0 macrophages or M1 macrophages were detached, fixed, and incubated with IgG from human serum (Sigma-Aldrich, Germany) for 20 min followed by incubation with anti-CD14 FITC human monoclonal antibody for 45 min at room temperature in the dark and washed twice with PBS. Samples were analyzed by flow cytometry using a flow cytometer (BD Biosciences, USA).

The surface markers of stimulated cells were detected by flow cytometry. Flow cytometry (FC) was used to evaluate the expression level of CD86 in macrophages after intervention in each group. Macrophages were collected after intervention and washed three times with PBS. The cell suspension of macrophages in each group was transferred into 1.5 ml EP tubes and incubated with blocking antibody CD16/32 (Biolegend, USA) for 10 min on ice. After washing twice, cells were incubated in PBS plus Intrapore Permeabilization reagent and the following antibodies (PE anti-human CD86, Biolegend, USA) on ice for 30 min in the dark. After washing twice, the cells were suspended in 500 μl PBS with 3% FBS and then detected by flow cytometry (BD Biosciences, USA).

To determine the apoptosis rate of chondrocytes in different groups, chondrocytes were stained with FITC-labeled Annexin V and propidium iodide (PI) using an Annexin V-FITC Apoptosis Detection Kit (Beyotime, China) according to the manufacturer’s guidelines. In brief, cells (5 × 10^5^ cells/well) were seeded in 6-well plates and treated as described above. After treatment, both suspended and adherent cells were harvested, washed with precooled PBS successively, and then resuspended in 100 μl binding buffer. Ten microliters of FITC-labeled Annexin V and 5 μl of PI were added to each tube, followed by 15 min of incubation in the dark. Subsequently, the samples were analyzed by flow cytometry (BD Biosciences, USA).

### Experimental design and treatment

The Ethics Committee of Affiliated Changzhou No.2 People’s Hospital with Nanjing Medical University approved all animal experiments. Rats were housed on a standardized pelleted diet and supplied with tap water. OA was induced in 8-week-old male SD rats (purchased from Changzhou Cavens Laboratory Animal Co., Ltd., China) by destabilized medial meniscus (DMM) surgery of the left knees as described [62]. To establish the OA animal model, the rats were anesthetized by intraperitoneal injection of pentobarbital (35 mg/kg) followed by destabilized medial meniscus (DMM) surgery on the left knee [56, 63]. Forty-eight rats were randomly divided into six groups (n=8 for each group): the sham group, the DMM group, the DMM+Vehicle group, the DMM+Rspo2 (20 μg/kg) group, the DMM+Ast (20 mg/kg) group, and the DMM+Ast (20 mg/kg) +Rspo2 (20 μg/kg) group. Eight rats in the sham group were used as the sham operation group in which the anterior cruciate ligament (ACL) was exposed but not transected. 1 week later, rats in each group were treated. The DMM+Rspo2 group and the DMM+Ast group were intra-articularly injected with 20 μg/kg rhRspo2 and 20 mg/kg astaxanthin, respectively. The rats in the DMM+Vehicle group were intra-articularly injected with the same volume of physiological saline and DMSO (vehicle). The rats in the DMM+Ast+Rspo2 group were intra-articularly injected with astaxanthin and Rspo2 at the same dose. The rats in the sham surgery group and DMM group did not receive any intra-articular injection. After the last treatment, the rats were euthanized and the knee joints were collected for further experiments.

### Histology assay

The collected cartilage tissue of the knee joint was fixed in 4% PFA for 24 hours and decalcified with 10% EDTA (pH=7.4) for 4 weeks. Then, the tissues were embedded in paraffin and sectioned to a thickness of 5 μm on the sagittal plane. The sections were then stained with hematoxylin-eosin (HE) and Safranin O. The degree of soft bone degeneration was scored according to the Mankin scoring standard.

### Statistical analysis

All experiments were independently performed in triplicate. All data analysis was conducted with the SPSS 26.0 statistical software package. The results are expressed as the means ± S.D.s. Differences between groups were assessed by unpaired two-tailed Student’s t-test and one-way ANOVA. *P* < 0.05 was considered statistically significant.

### Availability of supporting data

The datasets used and/or analyzed during the current study are available from the corresponding author on reasonable request.

## Supplementary Material

Supplementary Table 1
