# Supplementary material for: Astaxanthin prevents osteoarthritis by blocking Rspo2-mediated Wnt/β-catenin signaling in chondrocytes and abolishing Rspo2-related inflammatory factors in macrophages
Source: Aging (Albany NY). 2023 Jun 23;15(12):5775–97. doi: 10.18632/aging.204837 (PMC10333078; doi:10.18632/aging.204837)
Supplement: Supplementary Table 1 [file aging-15-204837-s001.pdf]

SUPPLEMENTARY TABLE

Supplementary Table 1. Primers used in this study.

Primer	Forward primer 5'-3'	Reverse primer 5'-3'
iNOS	CACCTTGGAGTTCACCCAGT	ACCACTCGTACTTGGGATGC
TNF-α	ACGGCATGGATCTCAAAGAC	TTGGTTGTAGAGGGCAAGGACCT
IL-1β	GCTGCTTCCAAACCTTTGAC	AGCTTCTCCACAGCCACAAT
Arg-1	CTCCAAGCCAAAGTCCTTAGAG	AGGAGCTATCATTAGGGACATC
IL-10	GCTCCTAGAGCTGCGGACT	TGTTGTCCAGCTGGTCCTTT
Rspo2	TGGCTCAGTGTGTGCTGAGAGAAT	AAGGTCACGAGTGAGTAGCGCATT
Col-2	TGGACGATCAGGCGAAACC	GCTGCGGATGCTCTCAATCT
Aggrecan	GGTGAACCAGTTGTGTTGTC	CCGTCCTTTCCAGCAGTC
Sox-9	AGGAAGCTCGCGGACCAGTAC	GGTGGTCCTTCTTGTGCTGCAC
MMP-1	ATGAAGCAGCCCAGATGTGGAG	TGGTCCACATCTGCTCTTGGCA
MMP-3	CACTCACAGACCTGACTCGGTT	AAGCAGGATCACAGTTGGCTGG
MMP-9	GTACCACGGCCAACTACGAC	GCCTTGGAAGATGAATGGAA
MMP-13	CCTTGATGCCATTACCAGTCTCC	AAACAGCTCCGCATCAACCTGC
IL-6	AGACAGCCACTCACCTCTTCAG	TTCTGCCAGTGCCTCTTTGCTG
VEGF	CCTGTGTGCCGCTGATG	CGCATGATCTGCATGGTGAT
GAPDH	CAA ATTCCATGGCACCGTCA	GACTCCACGACGTACTCAGC
